# Effect of chemical passivation on corrosion behavior and ion release of a commercial chromium-cobalt alloy

**DOI:** 10.34172/joddd.2020.037

**Published:** 2020-09-21

**Authors:** Elnaz Moslehifard, Tahereh Ghaffari, Samineh Mohammadian-Navid, Mina Ghafari-Nia, Amirali Farmani, Farzad Nasirpouri

**Affiliations:** ^1^Department of Prosthodontics, Faculty of Dentistry, Tabriz University of Medical Science, Tabriz, Iran; ^2^Private Practice, Tabriz, Iran; ^3^Faculty of Materials Engineering, Sahand University of Technology, Tabriz, Iran

**Keywords:** Chromium-cobalt alloy, Corrosion, Ion release

## Abstract

**Background.** Corrosion resistance and ion release of alloys play a crucial role in biomedical applications. The present study aimed to investigate an increase in corrosion resistance and reduction in ion release in a commercial Co-Cr-Mo alloy by the chemical passivation method.

**Methods.** Based on ADA97, 20 samples of Flexicast alloy were cast, surface-polished, and electrolytically passivated at room temperature for 24 h in a sodium sulfate solution. Corrosion and ion release of the alloys before and after passivation were studied in normal saline solution. Corrosion resistance and the ion release rates were measured by the weight loss method and atomic absorption spectroscopy, respectively, before and after passivation after 1, 2, 3, and 4 weeks. The surface morphology of the samples was examined using scanning electron microscopy (SEM). The results were analyzed with Kruskal-Wallis and Mann-Whitney tests using SPSS 20 at a significance level of <0.05.

**Results.** The corrosion rate in the passivated samples was significantly lower than the non-passivated samples at the intervals (1, 2, 3, and 4 weeks) (P<0.05). The passivation of the alloy significantly reduced Co and Cr ion release in the first and fourth weeks, and in the first, second, and fourth weeks, respectively (P<0.05). SEM images revealed localized pitting associated with the corrosion, which was less significant in passivated samples.

**Conclusion.** Chemical passivation of the CR-Co alloy significantly reduced corrosion and ionic release of Cr and Co over time.

## Introduction

Alloys are affected by humidity, temperature changes from zero to 70°C, and pH changes from 2 to 11 during the chewing process in the oral environment. Different foods have different pH levels, with most having a pH value of <7, which can be considered an aggressive environment.^1,2^ An alloy with low corrosion resistance releases more metal elements in the body and increases the risk of unwanted reactions with tissues.^3^ This phenomenon is a progressive process in the oral cavity, resulting in the release of elements.^4^ Inadequate quantities of such elements can cause inflammation in the periodontal tissues and oral mucosa. Although there is evidence of changes in immune responses in vitro, the role of these ions is unknown in inflammatory diseases including gingivitis and periodontitis.^5^

One of the essential factors in biological adaptation is the corrosion property of the alloy. The number of ions released as a result of corrosion depends on the alloy chemical composition and microstructure as well as the casting and polishing conditions.^2^ As a general rule, any dental alloy in the oral environment will be exposed to corrosion due to oral conditions. Therefore, alloys should possibly be used with minimal ion release and harmful ions. In addition to the release of ions, the amount of release and the duration of tissue contact with such elements influences the biological response to the alloy. Free ions can cause problems with the restoration function and its destruction, such as breakage of the solder and, from an aesthetic viewpoint, the tarnishing of metal surfaces will deform the restoration.^6^ However, specific conditions of oral environments such as saliva, dental plaque, bacteria, gastric acid reflux, and so on will affect the corrosion of alloys.^5,7^

Co–Cr-Mo alloys exhibit better biocompatibility and corrosion resistance than Ni–Cr-based alloys because Co, Cr, and Mo are considered less toxic than Ni and are released at much lower concentrations.^8^ A few reports are available on the cytotoxicity of Co-Cr-base alloys. In one study, the magnitude of metallic ions released from Co-Cr-Mo alloys was higher in the buffered saline solutions than in the studied salivary samples, though it remained within the physiological limit of trace elements in the human body.^9^ Another study demonstrated that a Co–Cr alloy induces cytotoxicity and inflammatory responses in the human body, which can be prevented via antioxidants. Co-Cr dental alloy induces cytotoxicity and inflammatory responses in human gingival fibroblasts and osteoblasts.^10^

Passivation of metal surfaces in the human body can improve the corrosion resistance of alloys. Passivation is a condition accompanied by the formation of a resistant layer on the surface, thereby reducing corrosion significantly. This results from the formation of a protective layer on the metal surface. Sodium sulfate was used as the passivation solution in which a passive layer composed of chromium-rich oxide was grown on Cr-Co alloys. The oxide film grown on the alloy and its wide application in the biomedical industry indicate that Co-Cr alloys are well known for their biocompatibility. Cr-rich passive oxide film, which is highly resistant to acid, contributes to the alloys’ biocompatibility.^11^ Alloys coated with such layers will have lower electrochemical potential.^12^ Passivation can be achieved by chemical reactions.^13^ Passive layers will have lower ion conduction, lower solubility, higher abrasion resistance, and higher adhesion.^14^ The biocompatibility of Co-Cr alloy is associated with high corrosion resistance, resulting from the formation of an internal passive layer on the alloy surface. The release of this layer or its local degradation can release ions into the oral cavity.^15^

Matkovic et al^16^ reported that the incorporation of Mo and Cr improved the corrosion resistance of alloys. Another study found that chromium depletion by <16% led to increased corrosion.^17^ Denizoğlu et al18 evaluated the release of ions from two base metal alloys at different pH levels (4, 5, and 7) and observed the highest and the lowest releases for Ni and Cr, respectively. They reported that pH changes significantly affected the overall release of cobalt metal ions but did not affect that of Ni or Cr. Also, the type of alloy did not differ in the release of elements. Rincic et al^19^ showed that pH values and immersion time affected the release of Co, Cr, Fe, Zn, and Ni ions from dental alloys. This theory has been confirmed that alloy surface composition is essential in the corrosion behavior and alloy release for noble metals and base metal. The alloy surface plays a vital role in low-pH solutions. For example, lack of surface polishing will increase porosity and corrosion, as shown by McGinley et al.^20^

No study has so far been conducted on the passivation of this alloy. Since this method might be effective in alloy corrosion according to similar previous studies, this study aimed to investigate the effect of chemical passivation on corrosion behavior and ion release of Flexicast Co-Cr alloy.

## Methods

In this in vitro study, 20 samples of Flexicast alloy (corrosion and ion release groups [n=10]) were prepared based on ADA97. Patterns with a diameter of 8 mm and a thickness of 1 mm were prepared using a plastic disk. The plastic pattern was sprued, invested with a phosphate-based investment (Deguvest L, Degudent GmbH, Rodenbacher, Germany), and the wax was removed according to the manufacturer’s instructions. The Flexicast alloy (American Dent - All, Glendale CA, USA) (Motorcast, Degussa, Germany) was cast using a centrifugal device (Motorcast, Degussa, Germany) with an O_2_ torch. The alloy consisted of Co (63%), Cr (29%), Mo (6.1%), Ni (<1%), and Si (<1%). After casting, the samples were polished to obtain a mirror plane using sandpapers (320, 400, 600, and 800 grits) (Sand Paper 991 A, Softflex, Wasserfest, Germany), a polishing wheel, and polishing paste (Bego, Bremen, Germany). Then, the samples were cleaned with deionized water for 10 min using an ultrasonic device (Ultrasonic Processor, Hielscher, Germany); 70% propanol and hot air (250°C) were used for final preparation of samples.^21^

The effect of passivation was investigated by the preparation of a chemical solution (Na2SO4.10H2O [pH=7] + graphite) based on the reference.^11^ The samples were placed in this solution for passivation at 20°C for 24 hours. The second series of the samples were inserted in a normal saline solution. An electrolyte (100 mL) was used for each corrosion study. After mounting the electrodes, electrochemical stability was achieved in 30 minutes. The solution temperature was maintained at 1.37°C to mimic the oral environment.^22,23^

In this method, the corrosion rate was measured based on the weight loss method by weighing the samples before and after corrosion. First, the dimensions of the samples were carefully measured to determine their surfaces and initial weights. The passivated group sample was first placed in a passivation solution (Na2SO4.10H2O [pH=7] + graphite). Then, all the samples of the two groups were placed in a normal saline solution (NaCl, 3.5 wt%) at room temperature. The samples were placed in a special container containing the solutions in different conditions for 1, 2, 3, and 4 weeks. Afterward, the samples were washed in an ultrasonic bath with acetone, ethanol, and isopropanol solutions, respectively, for 10 min, and then weighed carefully using an accurate scale (precision: 0.01 mg).

The corrosion rate was calculated based on the unit of weight/surface/time (mpy = 534 W/D.A.T), where mpy is mil (one-thousandth of an inch) per year, W is weight loss (mg), D is sample density (g/cm^3^), A is sample surface in a square inch (in^2^), and T is the test time (h).

To measure the release of Cr and Co ions, the samples were immersed in Na_2_SO_4_.10H_2_O (pH=7) + graphite solution for 24 h to passivate, and then retrieved from the solution and placed in a normal saline solution. Ion release was examined weekly for four weeks. To measure the release of Cr and Co ions, the samples were, prepared, degreased, and immersed in 20 mL of NaCl solution (3.5 wt%) in closed polyethylene containers at ambient temperature from one day to four weeks.^14^ The ions released in the solution were measured by an atomic absorption spectroscopy (AAS) device (model Nova AA400, Analytic Jena, Germany).

## Results

A comparison of corrosion in each group revealed that the corrosion rate was similar in non-passivated samples at the 4 study intervals (in 4 weeks). Corrosion rate was significantly different in passivated samples in 4 weeks (P<0.05). The corrosion rates were similar and significantly lower in the first and second weeks than the third and fourth weeks. Table 1 compares the corrosion rates in the two groups of Cr-Co samples with and without passive films. The data showed that the corrosion rate in passivated samples was significantly lower than the non-routine samples at each of the study intervals (1, 2, 3, and 4 weeks) (P<0.05).

**Table 1:** Comparison of corrosion rates in passivated and non-passivated samples in four weeks

**Weeks**	**N**	**Passivated**		**Non-passivated**		**P-value**
		**Mean**	**SD**	**Mean**	**SD**	
1	10	0.0045	0.0023	0.0155	0.0216	0.001
2	10	0.0070	0.0026	0.0213	0.0220	0.026
3	10	0.0113*	0.0037	0.0303	0.0218	0.014
4	10	0.0150*	0.0075	0.0354	0.0214	0.011
P-value*		0.000		0.0186		

P-value: Mann-Whitney test results, *P-value: Kruskal-Wallis test results, *Significance level

Significant differences were observed in Co and Cr ion release rates between passivated and non-passivated samples in 4 weeks (P<0.05) (Table 2). Co ion release rate in passivated samples was similar and significantly lower in the first and second weeks than the third and fourth weeks (P<0.05). Cr ion release rate in passivated samples was significantly lower in the first week than the other weeks (P<0.05), with a significantly higher ion release rate in the fourth week than the other weeks (P<0.05) and similar ion release rates in the second and third weeks. Cr ion release rate in non-passivated samples was significantly lower in the first and second weeks than the third and fourth weeks (P<0.05). In the first, second, and third weeks, Cr ion release was similar in non-passivated samples while it was significantly higher in the fourth week than the other weeks (Table 2).

**Table 2:** Comparison of Co and Cr ion release rates in passivated and non-passivated samples in four weeks

**Weeks**	Co					Cr				
	**Passivated**		**Non-passivated**		**P-value**	**Passivated**		**Non-passivated**		**P-value**
	**Mean**	**SD**	**Mean**	**SD**		**Mean**	**SD**	**Mean**	**SD**	
**1**	0.252	0.118	0.490	0.077	0.000	0.576	0.094	0.766	0.060	0.000
**2**	0.364	0.039	0.436	0.108	0.063	0.706*	0.013	0.808	0.048	0.000
**3**	0.724*	0.212	0.774*	0.160	0.814	0.714	0.011	0.722	0.044	0.583
**4**	0.961*	0.256	1.122*	0.058	0.043	1.236*	0.185	1.466*	0.203	0.017
P-value*	0.000		0.000			0.000		0.000		

P-value: Mann-Whitney test results, *P-value: Kruskal-Wallis test results, *Significance level

**Figure 1:**
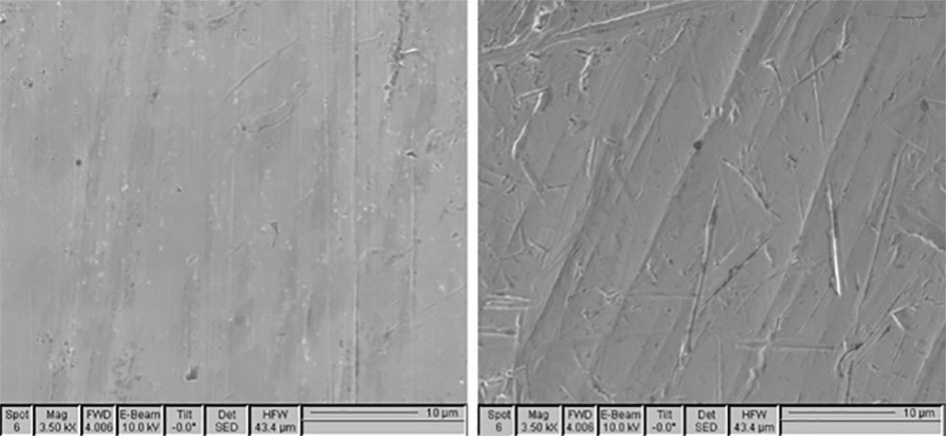
SEM images of CO‒Cr sample surfaces; left: passivated sample before immersion in normal saline solution, right: non-passivated sample.

**Figure 2:**
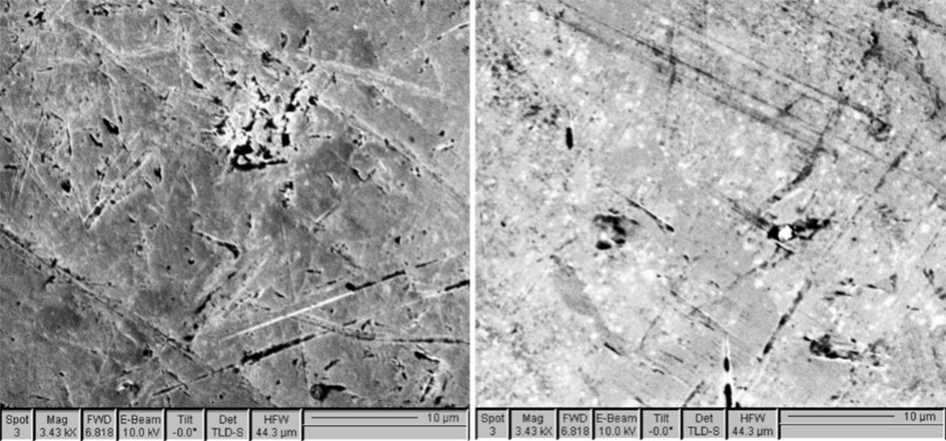
SEM images of CO-Cr sample surfaces; left: non-passivated sample after four weeks of immersion in normal saline solution, right: the passivated sample.

The surface morphology of Co-Cr samples showed that passivated samples had a smoother surface than non-passivated ones; in other words, there was less release in passivated samples than non-passivated ones (Figures 1 and 2).

## Discussion

The present study results showed that the corrosion rate of Flexicast CO-Cr base alloy at all four weeks (1, 2, 3, and 4 weeks) in the passivated samples was significantly lower than non-passivated samples; in other words, passivation reduced corrosion significantly over time. In non-passivated specimens, corrosion increased significantly in four weeks. Rylska et al^15^ demonstrated that passivation with Na_2_SO_4_ solution did not increase the electrochemical corrosion resistance of the alloy, but the electrolytic passivation in the same solution (Na_2_SO_4_) connected to a graphite cathode significantly increased corrosion resistance. In SEM studies of all the samples, the researchers found no perforation corrosion in the alloys after chemical inactivation; in other words, passivation increased the perforation corrosion resistance of alloys. Also, the examined samples showed no signs of cracking, which was also found in the present study.^15^ Na_2_SO_4_ is an indirect passivating agent, which is an alkaline compound that forms a high concentration of OH^−^ that reacts with H^+^ ions absorbed at cathode sites, eliminating them from the surface, thereby absorbing oxygen therein.^24^

Chromium content is one of the most critical factors affecting corrosion resistance. Therefore, an alloy containing the highest amount of chromium has a higher passivation potential.^25^ Wylie et al^26^ examined the effect of thermal treatment on microstructure, corrosion behavior, and cell response to two nickel-based alloys and found that Cr content was important in the corrosion resistance of nickel-based alloys at low pH values.

According to Matkovic et al,^16^ the incorporation of Mo and Cr into Co-Cr base alloys improved the corrosion resistance of alloys. In another study, corrosion increased with Cr depletion by up to 16%.^17^ The Flexicast Co-Cr base alloy used here contains Co (63%), Cr (29%), Mo (6.1%), Ni (<1%), and Si (<1%), with a high amount of Cr in the studied alloy. Ameer et al also showed that the Co-Cr-Mo alloy had higher corrosion resistance in artificial saliva than^27^ alloys containing Ni-Cr-Mo.

Surface inactivation or passivation of a metal reduces both cathode and anode reaction rates and prevents corrosion. Corrosion inhibitors are substances that reduce corrosion when they are added in small amounts. The mechanism of corrosion inhibitors is to interrupt cathode or anode reactions, or both. Passivators work by forming a thin, impermeable shell on the metal surface or interruption of either cathode or anode reactions.

In the present study, the alloy passivation significantly reduced Co and Cr ion release rates. Co ion release decreased in the first and fourth weeks, while Cr ion release decreased in the first, second, and fourth weeks. In non-passivated samples, Co and Cr ion release rates increased significantly over time, with a more significant effect of passivation on the reduction of Cr release rate than that of Co. SEM images also confirmed a lower release of passivated samples with a smoother surface than non-passivated samples, consistent with Rylska et al.^15^

It should be noted that an essential feature of the passivated layer formed on Cr is its very low release rate (about <0.1 µA/cm2). Passivated Cr does not show a fractured passive layer and local corrosion.20 Studies demonstrated that alloys containing 16‒27% Cr and 6‒17% Mo with no Be content had lower kerogen content, a homogenous protective oxide layer, and higher resistance to pitting.28,29 The Flexicast Co-Cr base alloy used here contains 63% Co, 29% Cr, 6.1% Mo, <1% Ni, and <1% Si, with a high amount of Cr in the studied alloy, and the results correspond to those of Huang et al and Roach et al.^28,29^

Briefly, several factors, including the chemical nature of elements, the metallurgical environment of elements, pH, pressure-related biomechanical conditions, pressure, and strain, surface quality and treatment, alloy abrasion, cleaning, and polishing, electrolyte composition, and the applied electrochemical potential, control the release of elements from alloys.^1,2^ In the present study, the samples were cleaned using sandpapers, polishing wheel, and polishing paste until obtaining a mirror surface and then were cleaned using an ultrasonic device and deionized water.

Denizoglu et al. evaluated the release of ions from two base metal alloys at different pH levels (4, 5, and 7) and observed the highest and the least releases for Ni and Cr, respectively. They reported that pH changes significantly affected the overall release of alloy and Co, but did not affect that of Ni or Cr. Also, the type of alloy did not differ in the release of elements.^18^

It should be noted that corrosion is affected by the environment and the metal type. The electrochemical reaction is also polarized or disturbed by environmental factors. Environment acidity has a significant effect on the passive layer formation and, consequently, corrosion. In a study by Dong et al,^30^ the corrosion behavior of dental alloys was investigated using a 7-day immersion test in a strong acid, a weak acid, and neutral water. Their results showed that Co-Cr alloy resistance was more corrosion resistant in the strong acid than in a weak acid and neutral water. In other words, the alloy had slight corrosion in an acidic environment due to the alloy inactivation by forming a surface protective oxide layer, which was the product of the thin brown corrosion present in the alloy, cobalt, and chromium oxide.

Previous studies on the Mo-Cr-Co alloy showed that 1) alloy samples were passivated by placing in sodium sulfate solution, and a layer of chromium oxide evenly covered the surface; 2) the corrosion mechanism in these alloys is of a local type associated with the formation of pitting; 3) corrosion and ion release rates decreased significantly by surface passivation operations.

Conclusion

Chemical passivation of the Flexicast Co-Cr alloy significantly reduced corrosion over time. At all the four time intervals, corrosion rates in the passivated samples were significantly lower than those in the non-passivated samples. The ion release rate in the passivated group was significantly lower than the non-passivated group. SEM images demonstrated higher levels of porosity and corrosion in the non-passivated group.

## Acknowledgments

This article was written based on a dataset from two DDS theses entitled “The effect of chemical passivation on the corrosion of a commercial cobalt‒chromium alloy” and “The effect of chemical passivation on the ion release in a commercial cobalt‒chromium alloy” registered at Faculty of Dentistry, Tabriz University of Medical Sciences (reference numbers 61178 and 61170).

## Authors’ Contributions

EM initiated, conceptualized, and supervised the research work. SM, MG, and AF prepared the samples and performed experiments with collaboration of EM, TG, and FN. All the authors have contributed to analyzing the data and writing the manuscript. All the authors have read and agreed to the published version of the manuscript.

## Funding

Not applicable.

## Competing Interests

The authors declare no competing interests with regards to the authorship and/or publication of this article.

## Ethics approval

This study was approved by the local ethics committee under the code IR.TBZMED.VCR.REC.1398.099

## References

[1] Schuster GS, Lefebvre CA, Wataha JC, White SN (1996). Biocompatibility of posterior restorative materials. J Calif Dent Assoc.

[2] Geurtsen W (2002). Biocompatibility of dental casting alloy. Crit Rev Oral Biol Med.

[3] O’Brien WJ (1997). Dental materials and their Selection.

[4] Fathi MH, Mortazavi VS (2004). Comparative evaluation of the effect of clinical procedures on the corrosion of four brand dental amalgams. J Dent Sch.

[5] Geis-Gerstorfer J (1994). In vitro corrosion measurements of dental alloys. J Dent.

[6] Sarantopoulos DM, Beck KA, Holsen R, Berzins DW (2011). Corrosion of CoCr and NiCr dental alloys alloyed with palladium. J Prosthet Dent.

[7] Ameer MA, Khamis E, Al-Motlaq M (2004). Electochemical behaviour of recasting Ni-Cr and Co-Cr non-precious dental alloys. Corros Sci.

[8] Moslehifard E, Ghasemzadeh S, Nasirpouri F (2019). Influence of pH level of artificial saliva on corrosion behavior and nickel ion release of a Ni-Cr-Mo alloy: an in vitro study. Anti corrosion Methods and Materials.

[9] El Sawy AA, Shaarawy MA (2014). Evaluation of metal ion release from Ti6Al4V and Co-Cr-Mo casting alloys: in vivo and in vitro study. J Prosthodont.

[10] Kim EC, Kim MK, Leesungbok R, Lee SW, Ahn SJ (2016). Co-Cr dental alloys induces cytotoxicity and inflammatory responses via activation of Nrf2/antioxidant signaling pathways in human gingival fibroblasts and osteoblasts. Dent Mater.

[11] Ramírez-Ledesma Ramírez-Ledesma, AL AL, Roncagliolo P, Álvarez-Pérez MA, Lopez HF, Juárez-Islas JA (2020). Corrosion Assessment of an Implantable Dental Co-Cr Alloy in Artificial Saliva and Biocompatibility Behavior. J Materi Eng Perform.

[12] Ries LAS, Da Cunha Belo M, Ferreira MGS, Muller IL (2008). Chemical composition and electronic structure of passive films formed on Alloy 600 in acidic solution. Corros Sci.

[13] Vafaeian S, Fattah-Alhosseini A, Keshavarz MK, Mazaheri Y (2016). The influence of cyclic voltammetry passivation on the electrochemical behaviour of fine and coarsegrained AISI 430 ferritic stainless steel in an alkaline solution. J Alloys Compd.

[14] Mutlu-sagesen L, Ergun G, Karabulut E (2011). Ion release from metal-ceramic alloys in different media. Dent Mater J.

[15] Rylska D, Sokołowski G, Sokołowski J, Łukomska-Szymańska M (2017). Chemical passivation as a method of improveing the electrochemical corrosion resistance of Co-Cr based dental alloy. Acta Bioeng Biomech.

[16] Matkovic T, Matkovic P, Malina J (2004). Effects of Ni and Mo on the microstructure and some other properties of Co-Cr dental alloys. J Alloys Compd.

[17] Espeik S (1977). Corrosion of base metal alloys in vitro. Acta Odontol Scand.

[18] Denizoğlu S, Duymuş ZY, Akyalçin S (2004). Evaluation of ion release from two base-metal alloys at various pH levels. J Int Med Res.

[19] Rincić N, Baucić I, Miko S, Papić M, Prohić E (2003). Corrosion behavior of the Co-Cr-Mo dental alloy in solutions of different Ph values. Coll Antropol.

[20] Mc Ginley EL, Coleman DC, Moran GP, Fleming GJ (2011). Effect of surface finishing conditions on the biocampatibility of a Nickel-Chromium dental casting alloy. Dent Mater.

[21] Dobrzanski LA, Reimann L (2011). Influence of Cr and Co on hardness and corrosion resistance CoCrMo alloys used on dentures. J Achiev Mater Manuf.

[22] Sarkar NK, Greener EH (1973). In vitro corrosion resistance of new dental alloys. Biomat Met Dev Art Org.

[23] Al Jabbari YS (2014). Physico-mechanical properties and prosthodontic applications of Co-Cr dental alloys: A review of the literature. J Adv Prosthodont.

[24] Takaichi A, Nakamoto T, Joko N, Nomura N, Tsutsumi Tsutsumi, Y Y, Migita S (2013). Microstructures and mechanical properties of Co-29Cr-6Mo alloy fabricated by selective laser melting process for dental applications. J Mech Behav Biomed Mater.

[25] Metikos-Hukovic M, Babic R (2007). Passivation and corrosion behaviours of cobalt and cobalt–chromium–molybdenum alloy. Corros Sci.

[26] Wylie CM, Shelton RM, Fleming GJ, Davenport AJ (2007). Corrosion of nickel-based dental casting alloys. Dent Mater.

[27] Ameer MA, Khamis E, Al-Motlaq M (2004). Electochemical behaviour of recasting Ni-Cr and Co-Cr non-precious dental alloys. Corros Sci.

[28] Huang HH (2002). Effect of chemical composition on the corrosion behavior of Ni-Cr-Mo dental casting alloys. J Biomed Mater Res.

[29] Roach MD, Wolan JT, Parsell DE, Bumgardner JD (2000). Use of x-ray photoelectron spectroscopy and cyclic polarization to evaluate the corrosion behavior of six nickel-chromium alloys before and after porcelain-fused-to-metal firing. J Prosthet Dent.

[30] Dong H, Nagamatsu Y, Chen KK, Tajima K, Kakigawa H, Shi S (2003). Corrosion behavior of dental alloys on version types of electrolyzed water. Dent Mater J.

